# An Account of Diseases in an Eastern District of London, from March 20, to April 20, 1808

**Published:** 1808-05

**Authors:** 


### *An Account of Diseases in an Eastern District of London, from March* 20, *to April* 20, 1808.

| ACUTE DISEASES. | |
|---|---|
| Peripneumonia Notha | 5 |
| Rubeola - - - | 3 |
| Ophthalmia - - - | 6 |
| Rheumatismus Acutus | 3 |
| CHRONIC DISEASES. | |
| Tussis - - - | 10 |
| Tussis cum Dyspnœa | 14 |

Phthisis

---

| | |
|---|---|
| Phthisis Pulmonalis - | 4 |
| Hæmoptysis - - | 2 |
| Ascites - - - | 7 |
| Hydrothorax - - | 8 |
| Dyspepsia - - | 5 |
| Hypochondriasis - | 3 |
| Paralysis - - | 2 |
| Dysuria - - - | 4 |
| Nephralgia - - | 1 |
| Amenorrhœa - - | 5 |
| Dysmenorrhœa - | 3 |
| Rheumatismus Chronicus | 15 |
| PUERPERAL DISEASES. | |
| Ephemera - - | 3 |
| Abcessus Mammæ - | 1 |
| Hæmorrhois - - | 4 |
| Menorrhagia Lochialis | 6 |
| INFANTILE DISEASES. | |
| Ophthalmia - - | 3 |
| Ophthalmia Purulenta | 1 |
| Herpes - - - | 3 |

The number of patients labouring under different species of dropsy, and referred to in the foregoing list, is uncommonly large. That particular species denominated hydrothorax has occurred in a number of instances. This disease frequently succeeds the different symptoms of pulmonic affection, and though it may exist for some time, and give rise to that difficulty of respiration which is a prominent feature in most of the diseases of the chest, yet it is not easily distinguished until some other symptoms appear. A dropsy of the chest is attended with difficult respiration, a short cough; a sense of weight and oppression, and an aggravation of these symptoms upon any sudden or quick motion. After some time the feet and ancles become œdematous, and the tumour gradually extends up the legs and thighs. This swelling increases in the evening, but in the course of the night it generally subsides, and in the morning the countenance appears bloated, and the upper extremities retain the impression of the finger. As the disease advances in its progress, the difficulty of breathing is increased by the patients lying in a horizontal posture; and, frequently, he is awakened by a sense of suffocation. Palpitation of the heart is another diagnostic symptom of hydrothorax. This is sometimes preceded by great irregularity and frequent intermission of the pulse, which symptoms are greatly aggravated by any unusual or sudden exertion. In this, as in other species of dropsy, a scarcity of urine is a pretty constantly attendant symptom, and in proportion as this can be relieved the other symptoms are less urgent and severe.

